# Prevalence of Dental Fluorosis and associated Risk Factors in Bagalkot District, Karnataka, India

**DOI:** 10.5005/jp-journals-10005-1373

**Published:** 2016-09-27

**Authors:** Taranatha Mahantesha, Uma B Dixit, Ramesh P Nayakar, Devasya Ashwin, Naveen K Ramagoni, Vijaya P Kamavaram Ellore

**Affiliations:** 1 Professor and Head, Department of Pedodontics and Preventive Dentistry, Navodaya Dental College & Hospital, Raichur, Karnataka, India; 2 Professor, Department of Pedodontics and Preventive Dentistry, Dr. D.Y. Patil Dental College & Hospital, Pune, Maharashtra, India; 3 Reader, Department of Prosthodontics, KLE Vishwanath Katti Institute of Dental Sciences, Belagavi, Karnataka, India; 4 Senior Lecturer, Department of Pedodontics and Preventive Dentistry, Kannur Dental College, Anjarakandy, Kerala, India; 5 Professor, Department of Pedodontics and Preventive Dentistry, Navodaya Dental College & Hospital, Raichur, Karnataka, India; 6 Reader, Department of Pedodontics, Navodaya Dental College & Hospital, Raichur, Karnataka, India

**Keywords:** Dental fluorosis, Fluoride, Fluorosis risk factors, Nutritional status.

## Abstract

**Introduction:**

An earlier epidemiological study by these authors revealed fluorosis at very low levels of fluoride concentrations in drinking water.

**Aim:**

The objective of present study was to investigate risk factors of dental fluorosis in permanent teeth in the villages of northern Karnataka, India.

**Materials and methods:**

The present survey was carried out in three villages of Hungund Taluk, Bagalkot District, Karnataka, India, with the fluoride concentration of 0.136, 0.381, and 1.36 ppm. Children aged between 9 and 15, with permanent teeth, were examined for dental fluorosis using Dean’s index, as per WHO criteria. Required relevant information regarding risk factors was obtained through a questionnaire.

**Statistical analysis:**

Data entry and analysis were performed using SPSS for Windows 16.0. Comparison of means of different indices by the three groups was performed using ANOVA and t-test (p < 0.05). Bivariate analysis was performed to identify significant risk factors that affected prevalence and severity of dental fluorosis. Those variables showing a statistically significant association (p < 0.05) on χ^2^ were entered into multiple logistic regressions to assess their independent effects.

**Results:**

In this study, we analyzed risk factors for both prevalence and severity of fluorosis. From multiple logistic regression analysis, only fluoride concentration in drinking water was found significant with prevalence of fluorosis and only nutritional status showed significant association with severity of fluorosis.

**Conclusion:**

Presence or absence of dental fluorosis in permanent teeth was significantly associated with fluoride concentration in drinking water. Once present, its severity was determined by nutritional status of the children - malnourished children exhibiting severe form of fluorosis.

**How to cite this article:**

Mahantesha T, Dixit UB, Nayakar RP, Ashwin D Ramagoni NK, Ellore VPK. Prevalence of Dental Fluorosis and associated Risk Factors in Bagalkot District, Karnataka, India. Int J Clin Pediatr Dent 2016;9(3):256-263.

## INTRODUCTION

Research on biology of fluorides has been very extensive during the last decades, especially after it was demonstrated in the 1930s that fluoride in drinking water prevents dental caries. Dean and others^1^ determined 1 ppm to be the optimal level of fluoridation for caries reduction while minimizing level of dental fluorosis. World Health Organization (WHO)^2^ reported that a level of approximately 1 ppm fluoride in temperate climates decreases level of dental caries to maximum extent without causing any harmful effects on the community. But steady increase in the prevalence of fluorosis has been reported in many countries despite guidelines recommended by WHO have been followed to optimally fluoridate their drinking water supply in order to decrease the prevalence of dental caries.^3,4^ A few studies have reported dental fluorosis at locations with very low levels of fluoride.^4-6^ Increase in dental fluorosis in industrialized countries has been mainly attributed to the intake of fluoride from other sources like fluoridated dentifrices, inadvertently prescribed fluoride supplements, and other topical fluoride agents.^3^

In India, use of fluoridated dentifrices, topical fluoride agents, and fluoride supplements are very minimal, especially in rural communities. There have been few studies reported in the literature which correlates relationship between fluoride concentration in water and fluorosis and investigate the indigenous risk factors causing dental fluorosis.^7,8^

It is known that fluoride content in drinking water is the primary cause of fluorosis. It is suggested that other factors like altitude of residence,^9^ climate,^10^ dietary habits,^11^ tea consumption,^12^ nutritional status of the child,^13,14^ duration of breast feeding,^15^ infant formulae,^16^ and use of fluoridated toothpaste^17^ have an influence on the prevalence and severity of dental fluorosis.

It is very difficult to isolate these factors and draw conclusions regarding the minimum level of fluoride concentration in drinking water which may prevent dental caries and will not cause dental fluorosis. The objective of present study was to investigate risk factors of dental fluorosis in permanent teeth in an Indian context.

## MATERIALS AND METHODS

The present survey was carried out in three villages of Hungund Taluk, Bagalkot District, Karnataka, India.

Base line water analysis data taken by the Zilla Panchayat Engineering Division, Bagalkot, was used for primary selection of villages. Initially 25 villages were selected. For water analysis, 500 mL water was collected from each of the respective sources of drinking water supplies in clean plastic bottles for fluoride estimation with the appropriate labeled information. Collected water samples were analyzed in the Department of Environmental Engineering, Basaveshwar Engineering College, Bagalkot. Analyses were performed with an ion-selective electrode (model 94-09) and a 720A meter (Orion, Beverly, USA) within a week of sample collection.

After the estimation of fluoride concentration in collected water samples, three villages were selected for the study with negligible, low, and near-to-optimal fluoride concentrations in their drinking water supply and with same altitude (531.69 m), average annual temperatures (29.46°C), average rainfall (63.72 mm), socioeconomic status, dietary pattern, and positive history of usage of same water supply for drinking purpose for at least 15 years.

The villages selected were Bevinal, Hirebadawadgi, Chittawadagi with 0.136, 0.381, 1.36 ppm of fluoride respectively.

The children aged between 9 and 15 years who were the life-time residents of the respective villages were selected for this study. A child was considered to be a continuous resident in this area if he/she had been born and lived all his/her life in that area except for short intervals, as during holidays, etc.

Permission to carry out the study was obtained from the District Education Officer, Bagalkot, and headmasters of all selected schools. Informed consent was obtained from children’s parents/guardians for participation of their children in the present study.

An elaborate questionnaire was designed to obtain relevant history of the child. To encompass all the objectives of the study, the questionnaire was divided into two parts. The first part collect the information regarding name, age, sex of the child, date of examination, village name, fluorosis index, and height and weight. Second part collect the information about oral hygiene habits (frequency, material used, introduction of brushing), amount of tea and water consumption per day, breastfeeding and weaning, food habits (type, frequency, staple diet), socioeconomic status of the parents, and general health of the child from birth to till date.

Prior to the commencement of the survey, calibration and standardization exercises for the examiner as well as patients were carried out with the help of photographs to minimize the subjective variation in different indices used in this study.

Clinical examination for all the subjects was carried out in the schools by single examiner and recording of data was done by a trained assistant, and both were blinded to the fluoride concentrations in the drinking water supply of the villages.

During the examination, a child was seated on an ordinary chair outside the school building. For the children who were absent in the school on the days of survey, clinical examination was carried out in their houses at the time of the interview. Indirect natural light was used for illumination. When necessary, dry cotton was used to remove debris. Then the child was asked to wet his/her teeth by tip of the tongue before recording the indices.

All the permanent teeth present in the child’s mouth were examined for dental fluorosis using Dean’s index, according to WHO criteria, without specially cleaning or drying the teeth. Each permanent tooth was given a score between 0 and 5.^18^ The final fluorosis index was based on the two most affected teeth. If they were different, lowest of the two was considered.

Intraexaminer reliability was calculated after reexam-ining 10% of the school children, during beginning of the survey and then 1 month thereafter.

Prior to oral examination, the data regarding name, age, and sex were entered from the school records. The houses of the children selected for the study were visited, and their mothers (if a mother was absent, then child’s father/ guardian) were interviewed by the examiner. Care was taken not to ask misleading questions and enough time was given for giving the most reliable account on the history.

Weight was measured by using spring loaded “Krups” weighing machine (Beliram and Sons, New Delhi), and height was measured by asking the child to stand erect and bare-footed and using a specially designed wooden scale attached with a fiber glass tape. A child’s height, weight, and age were used to determine his/her nutritional status using Waterlow classification.^19^ Using standard charts by National Center for Health Statistics (NCHS) for height, weight and age of boys and girls, percent weight for age, and percent weight for height were calculated for each child. Then the children were classified based on the Waterlow classification.^19^ For our study purposes, since fluorosis in permanent teeth is only affected by long-duration malnutrition, children who were normal and wasted but not stunted (short-duration malnutrition) were classified as well nourished, and stunted children (long-duration malnutrition) and stunted and wasted children (current and long-duration malnutrition) were classified as malnourished.

Total water intake per day of the age at examination was estimated from the number of glasses (250 mL) of water reported to be consumed per day. We had no possible way of directly measuring fluid intake during the period, most critical to the development of dental fluorosis (the first 6 years of life). However, water consumption data were recorded to throw some light on daily consumption of water in a tropical country like India, as well as to examine if it has any effect on dental fluorosis retrospectively as current water consumption may be assumed to be related to past water consumption per day.

## STATISTICAL ANALYSIS

Data entry and analysis were performed using SPSS for Windows 16.0. Comparison of means of different indices by the three groups was performed using ANOVA and t-test (p < 0.05). Bivariate analysis was performed to identify significant risk factors that affected prevalence and severity of dental fluorosis. Those variables showing a statistically significant association (p < 0.05) on χ^2^ were entered into multiple logistic regressions to assess their independent effects.

## RESULTS

For the purpose of analysis, three villages selected were divided into three groups as follows:

*Group I:* Children residing in area with fluoride concentration 0.136 ppm

*Group II:* Children residing in area with fluoride concentration 0.381 ppm

*Group III:* Children residing in area with fluoride concentration 1.36 ppm.

In the group I, 100 children were examined and 3 were excluded. In the group II, 100 children were examined and 1 child was excluded. In group III, 96 were examined and 3 were excluded. These children were excluded because they were not continuous residents of those particular villages.

Distribution of children in all the three groups by age, weight, and height is given in Table 1. No significant differences were found in age, height, or weight among the three groups.

Prevalence and severity of fluorosis in the groups I, II, and III are presented in the Table 2. For measuring severity of fluorosis, children with the Dean’s scores of 1 to 3 were considered to have mild fluorosis and those with scores 4 to 5 were considered to have severe fluorosis.^15^

Mean fluorosis for the groups I, II, and III were 0.12 + 0.47, 1.42 + 0.15, and 4.71 + 0.047 respectively and are presented in the Table 3. Mean fluorosis score of group I was found to be the lowest among the three groups, and the difference was significant. Mean fluorosis score of children in the group II was significantly lower than that in the group III.

**Table 1:** **Table 1:** Distribution of children in the groups I to III by age, height, and weight

		*Age (years)*				*Height (cm)*				*Weight (kg)*			
*Groups*		*Mean ± SE*		*Range*		*Mean ± SE*		*Range*		*Mean ± SE*		*Range*	
I (0.136 ppm)		11.38 ± 0.19		9-14.8		134.54 ± 1.22		109-166		27.88 ± 0.84		18-50	
II (0.381 ppm)		12.03 ± 0.12		9.6-14.7		141.29 ± 103		118-169		30.54 ± 0.77		19-55	
III (1.36 ppm)		11.8 ± 0.19		9.1-14.7		133.35 ± 1.23		111-163		27.68 ± 0.71		17-48	

**Table 2:** **Table 2:** Prevalence and severity of fluorosis

		*Dean’s index of fluorosis prevalence*														*Severity of fluorosis prevalance*			
*Groups*		*0*		*1*		*2*		*3*		*4*		*5*		*Total*		*Mild*		*Severe*	
I (0.136 ppm)		90 (92.7)		2 (2.1)		5 (5.2)		0		0		0		97		7 (7.3)		0	
II (0.381 ppm)		42 (42.4)		13 (13.1)		13 (13.1)		23 (23.3)		7 (7.1)		1 (1.0)		99		49 (49.5)		8 (8.1)	
III (1.36 ppm)		0		0		0		0		27 (29)		66 (71)		93		0		93 (100)	

Numbers in the parentheses indicate percentages

**Table 3:** **Table 3:** Mean (± SE) scores for Dean’s index of fluorosis by fluoride concentration in drinking water

*Groups*		*Number of children*		*Dean’s index for fluorosis Mean ± SE*	
I (0.136 ppm)		97		0.12 ± 0.047^a^	
II (0.381 ppm)		99		1.42 ± 0.15^b^	
III (1.36 ppm)		93		4.71 ± 0.047	

^a^Significantly lower than groups II and III (p < 0.001); ^b^significantly lower than group III (p < 0.001)

In this study we analyzed risk factors for both prevalence and severity of fluorosis separately. Total six risk factors, including fluoride concentration in drinking water, tea consumption, type of diet, nutritional status, breast-feeding duration, and water consumption per day, were analyzed to evaluate the correlation with the prevalence and severity of fluorosis.

In order to assess the associations between risk factors and prevalence of fluorosis, all 289 children were divided into two groups, namely, no fluorosis (Dean’s index 0) and presence of fluorosis (Dean’s index > 1). Bivariate analysis revealed positive associations between the prevalence of fluorosis and fluoride concentration in drinking water, frequency of tea consumption, nutritional status, and present water consumption (Table 4). When these positive risk factors were analyzed further by multiple logistic regressions to assess their independent association with prevalence of fluorosis, only fluoride concentration in drinking water was found significant (Table 5).

In order to find out the risk factors that associated significantly with severity of fluorosis, bivariate analysis was performed considering all 289 children and dividing them into two severity groups. Severity of fluorosis was determined to be either mild (Dean’s index: 1-3) or severe (Dean’s index: 4-5). Bivariate analysis between the risk factors and severity of fluorosis showed that fluoride concentration in drinking water, type of diet, and nutritional status had significant associations (Table 6). When these three risk factors were entered in multiple logistic regressions, only nutritional status showed significant association with severity of fluorosis (Table 7).

**Table 4:** **Table 4:** Bivariate analysis used to evaluate the correlation between prevalence of fluorosis and the risk factors

		*Prevalence of fluorosis*											
		*Dean’s index 0*				*Dean’s index ≥ 1*							
*Risk factors*		*No. of children*		*Percentage*		*No. of children*		*Percentage*		χ^2^		*p-value*	
*Fluoride concentration*													
0.136 ppm		90		92.78		7		7.22		165.37		0.000001	
0.381 ppm		42		42.42		57		57.58				S	
1.36 ppm		0		0		93		100					
*Tea consumption/day*													
None		38		55.88		30		44.12		6.84		0.032	
1 cup		46		49.46		47		50.54				S	
2 cups or more		48		37.5		80		62.5					
*Diet*													
Veg		51		42.85		68		57.15		0.65		0.421	
Mixed		81		47.64		89		52.36				NS	
*Nutritional status*													
Well-nourished		91		50.28		90		49.72		4.13		0.04	
Malnourished		41		38.0		67		62				S	
*Breastfeeding*													
<1 year		2		100		0		0					
1-2 years		128		45.7		157		54.3		2.41		0.300	
2 years or above		2		100		0		0				NS	
*Water consumption/day*													
4-7 cups		10		55.6		8		44.2		13.23		0.0013	
8-11 cups		100		51.8		93		48.2				S	
12 cups and above		22		28.2		56		71.8					

S: Significant; NS: Nonsignificant

**Table 5:** **Table 5:** Results of multiple logistic regression analysis performed to evaluate independent effects of risk factors on the prevalence of dental flurosis

		*Multiple logistic regression*							
*Risk factors*		*B*		*SE*		*p-value*		*Significance*	
Fluoride concentration		3.33		0.401		0.00001		S	
Tea consumption		0.141		0.224		0.528		NS	
Nutritional status		0.225		0.421		0.592		NS	
Water consumption		0.1189		0.388		0.759		NS	

S: Significant; NS: Nonsignificant

### DISCUSSION

A number of recent investigations^4,15^ performed in the industrialized countries have indicated that the prevalence of dental fluorosis is increasing even in less than optimally fluoridated communities. In India, definite dental fluorosis has been reported even at fluoride level of 0.4 ppm.^20^ In our previous study,^6^ we found fluorosis at low levels of fluoride concentration in drinking water supply. Hence, this study was undertaken to find out the risk factors of fluorosis in villages of north Karnataka, India. We selected three villages with 0.136, 0.381, and 1.36 ppm fluoride concentration in their drinking water supply.

**Table 6:** **Table 6:** Bivariate analysis used to evaluate the correlation between severity of flurosis and the risk factors

		*Severity of fluorosis*											
		*Mild (1-3)*				*Severe (4-5)*							
Risk factors		*No. of children*		*Percentage*		*No. of children*		*Percentage*		χ^2^		*p-value*	
*Fluoride concentration*												
0.136 ppm		7		7.2		0		0		127.03		0.000001	
0.381 ppm		49		49.5		8		8.1		S	
1.36 ppm		0		0		93		100					
*Tea consumption/day*													
None		12		17.6		18		26.6		0.315		0.854	
1 cup		16		17.2		31		33.3		NS			
2 cups or more		28		21.9		52		40.6					
Diet													
Veg		14		24.7		54		27.7		11.88		0.0006	
Mixed		42		11.8		47		45.4		S			
*Nutritional status*													
Well-nourished		45		28.86		181		62.63		18.88		0.00001	
Malnourished		11		10.2		56		51.8		S			
*Breastfeeding*													
< 1 year		0		0		0		0					
1-2 years		56		22.3		101		31.9		0.998		0.317	
2 years or above		0		0		0		0		NS			
*Water consumption/day*													
4-7 cups		2		11.1		6		33.3		5.36		0.068	
8-11 cups		40		20.7		53		27.5		NS			
12 cups and above		14		17.9		42		53.9					

S: Significant; NS: Nonsignificant

**Table 7:** **Table 7:** Results of multiple logistic regression analysis performed to evaluate independent effects of risk factors on severity of dental fluorosis

		*Multiple logistic regression*							
*Risk factors*		*B*		*SE*		*p-value*		*Significance*	
Fluoride concentration		22.90		98.14		0.81		NS	
Diet		–9.09		74.37		0.903		NS	
Nutritional status		1.73		0.834		0.038		S	

S: Significant; NS: Nonsignificant

Results from our study revealed that percentage of children with severe fluorosis increased with the increase in concentration of fluoride in drinking water. It was not unexpected that 100% of the children in the group III (fluoride concentration: 1.36 ppm) showed severe fluorosis. However, in the area with fluoride concentration 0.381 ppm, 50% of the children showed mild and 8% showed severe fluorosis. Surprisingly, in the area with 0.136 ppm fluoride concentration, only 7% children showed mild fluorosis.

Such a high prevalence was reported by many authors from different parts of world.^4,5^ They stated that apart from fluoride in the drinking water supply many other sources of fluoride are considered to be important in the development of dental fluorosis. In the industrialized countries, fluoride supplements,^15^ fluoride dentifrices,^16,17^fluoridated mouthwash,^15^ and infant formula^16^ are reported to be the major risk factors in the development of fluorosis.

In the developing countries, nutritional status, type of diet have been reported to be associated with increased risk for fluorosis even in the communities with less than optimal concentration of fluoride in drinking water.^11-14^Certain foods rich in fluoride, for example, tea, and locally grown food items in areas where the fluoride concentration in water used for irrigation is high may play an important role in development of fluorosis.^12,21,22^

As children residing in rural India, from where our study sample was taken, do not have access to fluoride supplements, fluoridated dentifrices, and fluoride mouthwash; these factors were not considered while evaluating the risk factors. In our sample, most of the children used finger and charcoal for tooth-cleaning. Out of 289 children examined, only 17 (6%) used toothbrush and toothpaste, that too very irregularly.

In the light of above background, we decided to evaluate frequency of tea consumption, type of diet (vegetarian or mixed), nutritional status, duration of breastfeeding, and present water consumption in addition to fluoride concentration in drinking water as risk factors associated with fluorosis.

In our study, fluoride concentration in drinking water was the sole determinant of the presence or absence of fluorosis. There is an interesting fact that two of the three groups included in our study had very low levels of fluoride concentrations in drinking water, much lower than the recommendation given by WHO.

In order to find out optimum level of fluoride concentration for this area, we used an equation proposed by Villa et al^23^ to calculate optimum level of fluoride concentration in drinking water supply in developing countries depending on the temperature, as temperature determines the daily consumption of water.

The equation is as follows:

Optimum fluoride concentration (mg/L)

= (0.022/0.56)/[0.0104 + (0.000724 × AMMT)],

where AMMT stands for annual mean maximum temperature in degree Celsius.

By using this equation, we substituted AMMT = 29.46 for the area of our study, which gave the optimum fluoride concentration for the area of our study to be 0.3886 mg/L. This calculated concentration was almost equal to the fluoride concentration in drinking water of group II in our study, where 58% of children exhibited dental fluorosis.

This finding indicates that the children in our study are ingesting appreciable amounts of fluoride from sources other than drinking water. We also found that fluoride concentration in drinking water did not have an independent effect on severity of fluorosis. This indicates that presence or absence of fluorosis was determined by fluoride concentration in drinking water, but once present, and if it was manifested as mild or severe fluorosis, did not depend on the fluoride in drinking water.

In the part of Karnataka where we conducted our survey, tea is the main beverage consumed by children and adults alike. A study conducted in villages of China reported prevalence of dental fluorosis from 52 to 84% in the area with fluoride concentration ranged from 0.11 to 0.32 mg/L.^12^ The study stated that tea consumption played a major role in the development of dental fluorosis.

In our study, we did not find tea consumption as an independently significant risk factor in the development of dental fluorosis. This is in correlation to the studies conducted in India.^7,8^ In our study most of the children used one or two cups of tea with milk and sugar per day. The studies that have reported positive association between dental fluorosis and consumption of tea include populations who drink black tea and in larger quantities. Since fluoride content of the tea consumed by these children was impossible to be measured, owing to its retrospective nature, we cannot comment on daily consumption of fluoride through tea by these children. However, it has been reported that fluoride content of tea decreases when milk and sugar are added in it.^21^

In our study, diet did not show independent effect on either prevalence or severity of fluorosis. This is in contradiction to the findings reported by Awadia et al.^11^They reported from a study conducted on children between age 6 and 18 years that the risk of developing dental fluorosis was seven times higher among nonveg-etarians than among vegetarians. Such a significant effect of diet in their study may be due to presence of already excess amount of fluoride in the drinking water supply (3.6 mg/L). In our study, groups I and II had negligible fluoride concentrations in their drinking water. Also, perhaps consumption of fluoride from either vegetarian or mixed diet was not significantly different, since children on mixed diet consumed more vegetarian food with occasional nonvegetarian food.

It has been suggested that nutritional status plays important role in the increase in prevalence of dental fluorosis.^13,14^ In our study, nutritional status was assessed from percent weight/age and percent weight/height. This is an accepted method.^19,24^ Data from the NCHS, approved by WHO, was used as a reference to calculate nutritional status, because the data from Indian Council of Medical Research (ICMR) has not widely used by researchers as the data are not representative of average Indian children.^24^

Our study showed that malnutrition is prevalent in this area of South India which is in agreement with study conducted by Kodali et al.^25^ Thirty-eight percent of the total children examined showed long-duration malnutrition. More than 62% of malnourished children presented with fluorosis (10% with mild, 52% with severe); however, only 49.72% well-nourished children were affected by fluorosis (29% with mild, 63% with severe). This is in agreement with the studies conducted in India and around the world.^13,14^

In our study, nutritional status showed an independent effect on the severity of fluorosis, malnourished children being more affected by severe fluorosis. Presence or absence of fluorosis was not affected by nutritional status on multiple logistic regression analysis.

It has been reported that inadequate dietary intake of calcium, usually due to inadequate milk consumption, has been associated with increased fluoride-related bone changes.^26^ Similar calcium deficiency during the formation and mineralization of teeth will lead to hypocalcification. In the children, we examined, fluoride concentration in drinking water was the major factor in deciding presence or absence of dental fluorosis. It may be postulated that the process of hypoplasia of teeth due to fluorosis was enhanced by children’s malnutrition, making malnourished children experience more of severe fluorosis.

Breastfeeding has been reported to be associated with prevalence of fluorosis.^15^ Long duration of breastfeeding postpones the consumption of water with high fluoride concentration. Children, when breast-fed, have breast milk as their primary daily intake of fluid. Breast-milk is shown to contain negligible amount of fluoride even in mothers who consume highly fluoridated water.^27^ This means that till the age the child is breastfed, he/she will not be affected by high fluoride in water or food.

Our study did not reveal any effect of breastfeeding duration on prevalence or severity of fluorosis. Most of the children that we examined fell into the group of duration of 12 to 24 months. Hence, statistical significance (either positive or negative) in our study is questionable.

In order to make any conclusive remarks on breastfeeding duration as a risk factor in the development of dental fluorosis, further study needs to be carried out with larger sample size.

It has been reported that children living in high temperature areas consume more water, and consumption of water in turn is associated with prevalence of fluorosis.^10^

Our study revealed that most of the children in the area we surveyed consumed more than 2 L of water per day. Daily water consumption of these children is much higher than that reported in various studies from different countries, performed on the children in the similar age group.^8,11,28^ We tried to do a retrospective association between present water consumption and dental fluorosis in permanent teeth. The results showed a significant association in bivariate analysis, but no significant association was found in the multivariate analysis.

## CONCLUSION

- Prevalence of dental fluorosis at fluoride concentration of 1.36 ppm was significantly higher than that at 0.136 and 0.381 ppm. Prevalence of dental fluorosis at fluoride concentration of 0.381 ppm was significantly higher than that at 0.136 ppm.
- Presence or absence of dental fluorosis in permanent teeth was significantly associated with fluoride concentration in drinking water.
- Once present, its severity was determined by nutritional status of the children, malnourished children exhibiting severe form of fluorosis.
